# 12-Year-Old Girl Diagnosed With Li-Fraumeni Syndrome and Concomitant Adrenocortical Carcinoma (ACC)

**DOI:** 10.7759/cureus.30836

**Published:** 2022-10-29

**Authors:** Umberto M Donato, Diego Torres, Andrew Galligan

**Affiliations:** 1 Pediatric Hematology Oncology, University of South Florida Morsani College of Medicine, Tampa, USA; 2 Medicine, Universidad de Puerto Rico, San Juan, PRI; 3 Pediatric Oncology, Moffitt Cancer Center, Tampa, USA

**Keywords:** pediatric oncology surgeon, tp53 mutations, li-fraumeni, pediatric solid tumours, s: adrenocortical carcinoma (acc)

## Abstract

Adrenocortical carcinomas (ACC) are classical presentations of germline cancer predisposition syndromes such as the Li-Fraumeni syndrome (LFS). Li-Fraumeni syndrome is a multiple cancer syndrome caused by germline alterations of the tumor protein 53 (TP53) tumor suppressor gene and is often associated with ACC. If minor adrenocortical tumors (ACTs) are detected early, resection has proven to provide patients with better outcomes. However, non-functioning ACCs are particularly insidious since these patients present late and with distant metastases. We present the case of a 12-year-old female with a history of Li-Fraumeni syndrome (LFS) and a non-hormone-secreting ACC in the context of an exceedingly rare c.743G>A (p.Arg248Gln) p53 mutation.

## Introduction

Adrenocortical malignancies, specifically adrenocortical carcinomas (ACC) are exceedingly rare, accounting for nearly .02% of all cancer-related mortalities worldwide [1]. The incidence of these neoplasms in the general population is approximately one case per 1,000,000 people per year and an increased prevalence in women than in men [2]. Patients with ACCs typically become symptomatic due to hormonal excesses (Cushing’s syndrome) and abdominal pain/discomfort. Patients with ACC of all stages have a five-year survival rate of 37% [3]. Moreover, certain ACCs that exceed defined histopathological parameters, such as a Ki-67 index > 10%, have an even more negative prognostic weight [2].

Adrenocortical carcinomas in pediatric individuals (<15 years of age) are even rarer compared to the general population. The ACC incidence in this subgroup is limited to 0.3 cases per million per year [4]. Furthermore, the overall five-year survival of pediatric ACC cases is better than in adult patients with a rate of approximately 50% [5-6]. Along these lines, childhood ACC is a classic presentation of neoplasms in germline mutations such as Li-Fraumeni syndrome (LFS). Adrenocortical carcinomas are a hallmark feature of LFS, a multiple cancer syndrome caused by germline alterations of the protein (p)53 tumor suppressor gene [7]. This particular gene encodes the p53 transcription factor responsible for maintaining genome integrity and stimulating apoptosis to limit uncontrolled cellular growth.

Certain p53 mutations have an increased risk of developing certain cancers. For example, in a municipality in Brazil, a germline tumor protein (TP) 53 mutation (R337H) is said to be responsible for a 15-fold prevalence of adrenocortical tumors (ACTs) in comparison to the worldwide populace [8], however, this does not rule out the role of environmental factors in generating this disparity. Nevertheless, the aforementioned R337H Tp53 mutation is said to be found in 95% of children diagnosed with adrenocortical tumors in southern Brazil [8]. If small ACTs are detected early, curative resection is more likely [8]. However, non-functioning ACCs (those that do not affect adrenal hormone production) are particularly insidious since these present late and with distant metastases [9].

## Case presentation

**Figure 1:**
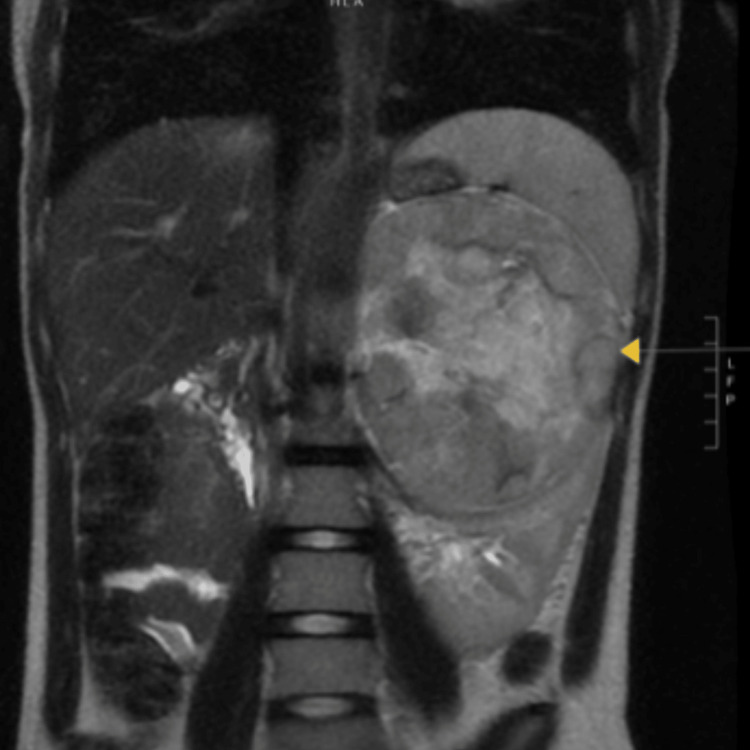
The full body MRI of the patient Yellow arrow points to a 13.9 cm necrotic left adrenal mass

**Figure 2:**
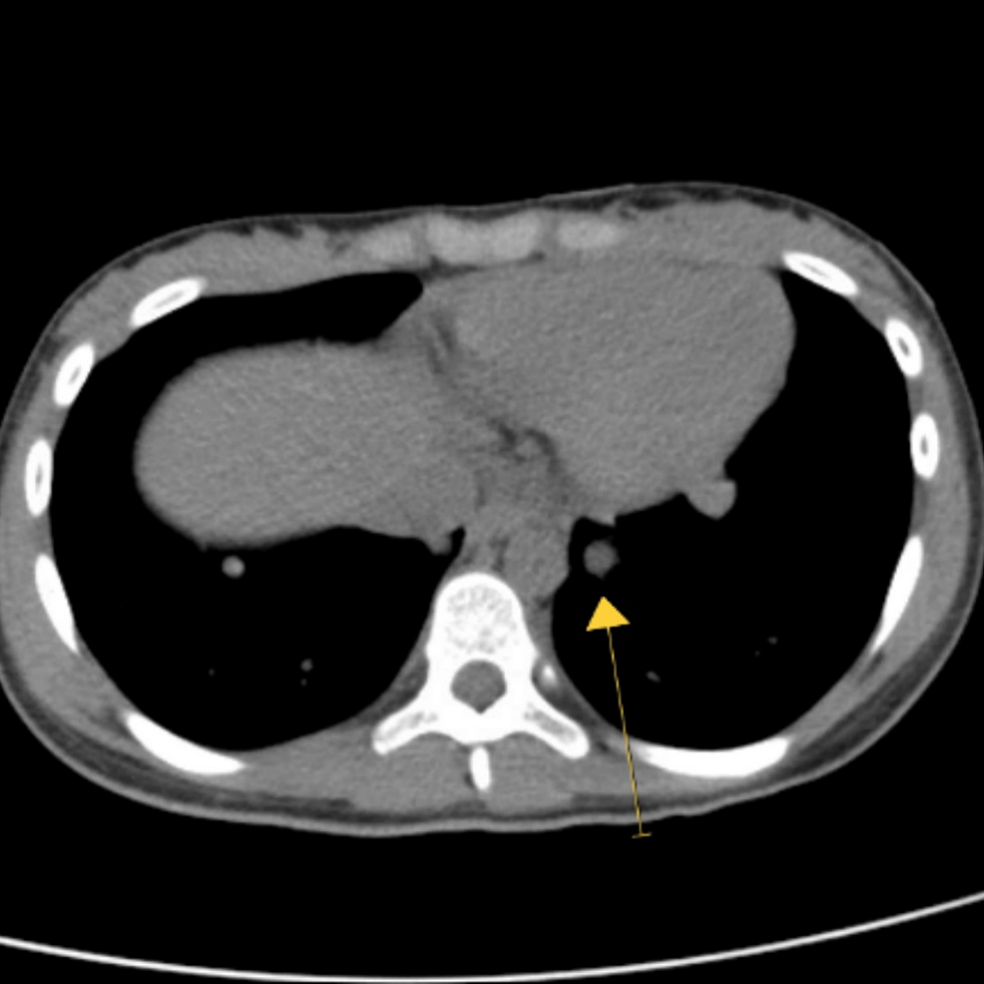
Chest CT scan of the patient Yellow arrow points to multiple bilateral pulmonary nodules measuring up to 1 cm in the left lower lobe

A 12-year-old female presented to her primary care provider (PCP) for routing imaging screening due to a history of LFS, specifically a pathogenic c.743G>A (p.Arg248Gln). The mother also had an oncologic history remarkable for a breast cancer diagnosis in the setting of the same LFS TP53 mutation. During a routine abdominal ultrasound screening, the PCP saw a suspicious heterogenous and metastatic solid mass and referred the patient for a total body MRI (the previous screening four months prior was negative for abnormalities). Urinalysis and adrenal hormone levels were within reference values for the patient's age. The MRI however, was notable for a 13.9 cm necrotic left adrenal mass highly suspicious for a metastatic non-functioning ACC in this patient with a reported history of LFS (Figure 1), two hepatic lesions measuring up to 1.2 cm, and an additional chest CT scan evidenced multiple bilateral pulmonary nodules measuring up to 1 cm in the left lower lobe that was concerning for metastases as well (Figure 2). Due to the concerns brought forth by the imaging results, the patient then underwent CT guided needle biopsy of the liver lesions and a CT-guided core biopsy of the adrenal mass followed by left adrenalectomy.

The results of the adrenal mass biopsy were consistent with a stage IV malignant/metastasizing adrenal cortical mass. The mass fulfilled the entirety of the Wernicke criteria of malignancy for pediatric ACTs including the two most significant markers for malignancy: venous and capsular invasion [10]. Moreover, these biopsy results in conjunction with the previous imaging showing multiple hepatic and lung lesions, provided supporting evidence that the ACC (the primary tumor) had metastasized to both the liver and the lung. These two sites are the most frequent metastatic areas for this type of primary tumor with 40% to 80% of these neoplasms spreading to the liver and lung [11].

Considering the aforementioned elements, the patient was started on the standard stage IV ACC ARAR0332 protocol with an imaging evaluation pending after three cycles of this chemotherapy. For this protocol, cycles are 21 days i.e., all 21 days of the regimen would include mitotane. The first five days of the protocol included the following drugs: cisplatin (days 1 to 2), etoposide (days 1 to 3); and doxorubicin: (days 4 to 5). The imaging evaluation (chest CT) revealed slight progression of the pulmonary metastases (growing in size) and thus the decision was made to switch to second-line treatment for ACC: pembrolizumab + mitotane (750 mg four times daily). This regimen consisted of one dose of pembrolizumab every 21 days with a continuation of mitotane daily. The patient tolerated these protocols well and is set to continue with the chemotherapy and routine imaging to monitor metastatic disease. 

## Discussion

An ACC is a rare malignancy that can affect any age group. Routine screening for LFS patients includes ultrasound examinations of the abdomen and pelvis every three months, complete urinalysis every three months, and blood tests every three months for beta-human chorionic gonadotropin (b-HCG), alpha-fetoprotein (AFP), 17-hydroxyprogesterone (17-OH-progesterone), testosterone, dehydroepiandrosterone sulfate (DHEAS), and androstenedione. Functioning ACCs present an overproduction of aldosterone, estrogen, testosterone, and cortisol that may result in Cushing’s syndrome or virilization [9]. For pediatric patients, virilization is the most common symptom (84.2%), including the emergence of pubic hair, hypertrophy of the clitoris, enlargement of the penis, hirsutism, and growth development. In contrast, non-functioning ACCs lack hormonal dysregulation, and patients are asymptomatic or present with non-specific symptoms such as abdominal pain and fatigue [9]. A recent retrospective study in Finland suggested that 80% of ACC patients studied in a period from January 2002 to February 2018 were asymptomatic and had an incidentally discovered mass [12], stressing the importance of early carcinoma detection for non-functioning ACC patients. Furthermore, familial LFS screening has been effective for the early detection of ACCs in pediatric patients [12-13]. For the case at hand, the patient had a family history of LFS and the routine ultrasound abdominal screening first detected the possible non-functioning ACC. Pediatric ACCs have been significantly linked to LFS and other syndromes such as familial adenomatous polyposis (FAP). Recent studies suggest a 50% to 80% prevalence of TP53 mutations in children and high germline TP53 positivity (58% for children <12 years old and 20% for children from 12 to 20 years old) [14]. Specifically, within TP53 mutations, high penetrance alleles (R175H, G245S, R248Q) and low penetrance alleles depict how variable penetrance plays a role in determining the risk for multiple primary malignancies [14]. For this case, the patient had a pathogenic c.743G>A LFS mutation and concomitant ACC. High penetrance is associated with germline mutation [14]. This case underlines the c. 743G>A mutation as a high penetrance allele for LFS and a plausible risk factor for multiple primary malignancies in accordance with the patient’s LFS family history. Although a previous study described a pediatric female that shared the same c.743G>A LFS mutation and concomitant ACC [15], in comparison to the available literature, this case uniquely presents a non-functioning ACC.

## Conclusions

This case serves to highlight the importance of routine and frequent cancer screening in Li-fraumeni patients. Specifically, we presented a rare pediatric pathogenic c.743G>A LFS mutation. Due to the rarity of this mutation and a non-functional ACC in the setting of pediatric LFS, this case contributes to the scarce literature covering the different oncologic intricacies of this pathology.
